# Identification of eQTLs associated with lipid metabolism in *Longissimus dorsi* muscle of pigs with different genetic backgrounds

**DOI:** 10.1038/s41598-020-67015-4

**Published:** 2020-06-17

**Authors:** Lourdes Criado-Mesas, Maria Ballester, Daniel Crespo-Piazuelo, Anna Castelló, Ana I. Fernández, Josep M. Folch

**Affiliations:** 1 Departament de Genòmica Animal, Centre de Recerca en Agrigenòmica (CRAG), CSIC-IRTA-UAB-UB, Barcelona, Spain; 2 Departament de Genètica i Millora Animal, Institut de Recerca y Tecnologia Agraroalimentàries (IRTA), Caldes de Montbui, Spain; 3 Departament de Ciència Animal i dels Aliments, Facultat de Veterinària, UAB, Bellaterra, Spain; 4 Departamento de Mejora Genética Animal, Instituto Nacional de Investigación y Tecnología Agraria y Alimentaria (INIA), Madrid, Spain

**Keywords:** Gene expression, Genome-wide association studies

## Abstract

Intramuscular fat content and its fatty acid composition affect porcine meat quality and its nutritional value. The present work aimed to identify genomic variants regulating the expression in the porcine muscle *(Longissimus dorsi)* of 45 candidate genes for lipid metabolism and fatty acid composition in three experimental backcrosses based on the Iberian breed. Expression genome-wide association studies (eGWAS) were performed between the muscle gene expression values, measured by real-time quantitative PCR, and the genotypes of 38,426 SNPs distributed along all chromosomes. The eGWAS identified 186 eSNPs located in ten *Sus scrofa* regions and associated with the expression of *ACSM5*, *ACSS2*, *ATF3*, *DGAT2*, *FOS* and *IGF2* (FDR < 0.05) genes. Two expression quantitative trait loci (eQTLs) for *IGF2* and *ACSM5* were classified as *cis*-acting eQTLs, suggesting a mutation in the same gene affecting its expression. Conversely, ten eQTLs showed *trans*-regulatory effects on gene expression. When the eGWAS was performed for each backcross independently, only three common *trans*-eQTL regions were observed, indicating different regulatory mechanisms or allelic frequencies among the breeds. In addition, hotspot regions regulating the expression of several genes were detected. Our results provide new data to better understand the functional regulatory mechanisms of lipid metabolism genes in muscle.

## Introduction

Studies on the traits that determine the quality of pork meat and their derived products have received increasing attention in recent years. The intramuscular fat (IMF) content and its fatty acid (FA) composition are considered determinant for meat quality, playing a central role in the nutritional values of the meat^1^. IMF influences meat flavour, juiciness, tenderness and firmness, which are important traits for consumer acceptance. On the other hand, its FA composition will determine how healthy is the product since it is well-known that some FAs are essential for humans, such as ω-3 and ω-6 polyunsaturated FAs (PUFAs)^2^.

During the last years, pig breeding companies have produced commercial pigs that grow faster and have superior carcasses. However, these carcasses have become leaner having less IMF and, therefore, producing a decrease in the meat quality according to consumers. Otherwise, local breeds such as the Iberian pig present a high-fat deposition and FA desaturation values and have a special interest in the production of high-quality dry-cured cuts, such as loin and ham^3^. Often the Iberian pig is crossed with other breeds to improve its reproductive and growth traits, although crossing has been associated with a decrease in meat quality^4^.

Several studies agree that genetic factors can determine intramuscular FA composition in pigs^1,5–7^. For example, significant breed effects have been reported for IMF, water binding capacity, colour, and tenderness. Thus, differences according to the genetic background have made the industry aware of it when improving the meat quality of pork^8^.

In recent years, genome-wide association studies (GWAS) have been used to detect genetic variants involved in FA composition traits, unravelling the complex genetic basis of these quantitative traits^9–14^. In general, genes involved in pathways or functions related to lipid metabolism are regulated at the transcriptional level, and studies conducted on the molecular mechanisms controlling these functions help to understand the genetic basis of traits related to FA composition in muscle tissue^15^. In previous studies, we have identified differentially expressed (DE) genes in the muscle transcriptome among two groups of extreme animals for FA composition in an Iberian x Landrace cross by RNA-Seq, reinforcing the view that variation in gene expression and its genetic basis may play an important role in the genetic determinism of these traits^16^. In addition, a genome-wide association study (eGWAS) of 45 lipid-related genes in the muscle of 114 Iberian × Landrace animals allowed the identification of genomic regions regulating the expression of these genes^17^. Two other gene expression studies related to lipid metabolism were performed in liver and backfat in the same experimental population^18,19^. However, the identified eQTLs have not been validated in other genetic backgrounds.

The main goal of the present study was the identification of genomic variants regulating the expression in pig muscle of 45 candidate genes for lipid metabolism and fatty acid composition. To achieve this objective, an eGWAS study was conducted for 355 animals using gene expression data of 114 BC1_LD pigs (25% Iberian and 75% Landrace) previously generated in Puig-Oliveras *et al*. (2016)^17^, and re-analysed in the present study using the *Sus Scrofa* 11.2 genome assembly, and new expression data of 122 BC1_DU (25% Iberian and 75% Duroc) and 119 BC1_PI (25% Iberian and 75% Pietrain) pigs.

## Results and discussion

### Sex and genetic background effect on gene expression

A sex bias in the expression of genes associated with lipid metabolism has been previously described in muscle and other tissues such as liver^17–21^. Hence it is relevant to understand the mechanisms of sex-differential gene expression.

**Figure 1:**
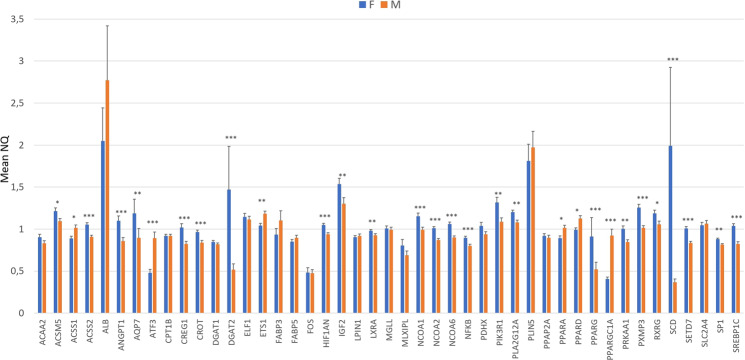
Comparison between females (F) and males (M) of mRNA expression levels of 45 lipid-related genes in animals from the 3BCs. Data are presented as mean ± standard error of the mean (SEM). Significant differences are labelled as *p-value ≤ 0.05, **p-value ≤ 0.01 and ***p-value ≤ 0.001.

In the global study, including the three backcrosses (3BCs), 30 out of the 45 genes presented significant sex effect (*p*-value ≤ 0.05) on gene expression: *ACSM5*, *ACSS1*, *ACSS2*, *ANGPT1*, *AQP7*, *ATF3*, *CREG1*, *CROT*, *DGAT2*, *ETS1*, *HIF1AN*, *IGF2*, *LXRA*, *NCOA1*, *NCOA2*, *NCOA6*, *NFKB*, *PIK3R1*, *PLA2G12A*, *PPARA*, *PPARD*, *PPARG*, *PPARGC1A*, *PRKAA1*, *PXMP3*, *RXRG*, *SCD*, *SETD7*, *SP1* and *SREBP1C* (Fig. 1). In general, there were more genes over-expressed in females, 24 out of 30, than in males. Six genes presented higher expression in males: *ACSS1*, *ATF3*, *ETS1*, *PPARA*, *PPARD* and *PPARGC1A*, being some of them relevant regulators implicated in lipolytic pathways. Genes over-expressed in females were implicated in transcriptional regulation and control (*CREG1*, *LXRA*, *NFKB1*, *NCOA1*, *NCOA2*, *NCOA6, PPARG, PRKAA1*, *RXRG*, *SP1* and *SREBP1c*), FA β-oxidation (*CROT*, *PXMP3* and *SCD*), lipid storage (*ACSM5*, *DGAT2*, *HIF1AN* and *AQP7*), cholesterol (*ACSS2*, *ANGPT1* and *SETD7)* and the AKT pathway (*IGF2*, *PIK3R1* and *PLA2G12A*). In addition, the *IGF2* gene, which has been involved in muscle growth and fat deposition^22^, showed a higher expression in females.

Overall these results are in accordance with previous studies describing differences in fat distribution and lipid metabolism between males and females^23,24^. Among the list of sex-biased genes, it is worth to highlight the role of *SREBP1C* and the *PPARs* members as key regulatory genes for lipid metabolism. In humans, males tend to present higher activity in lipolytic pathways, with a lower risk to gain fat and develop obesity than females^23^. In our study, *PPARA*, *PPARD*, and *PPARGC1A* presented higher expression in males than in females. Differences in PPARA activation between sexes seems to be influenced by the female hormone estrogen which has been shown to inhibit the PPARA action in the liver of mice^25^. Both in human and rodent skeletal muscle, *PPARA* and *PPARD* expression induce genes involved in fatty acid import and oxidation, increasing lipid oxidation and decreasing triacylglycerol accumulation. In addition, *PPARD* have been shown to increase glucose uptake and prevent insulin resistance^25^. Finally, *PPARGC1A* is a transcriptional co-activator that cooperates with *PPARA* to promote mitochondrial oxidative metabolism^26^. On the contrary, human females present higher rates of lipogenesis and accumulation of triglycerides, so they have a higher risk to gain fat and develop obesity^23^. In a similar way, female pigs seem to develop obesity more readily than male pigs^24^. In this regard, the higher expression of *SREBP1C*, a transcription factor that regulates the expression of a broad range of lipogenic genes^27^ such as *SCD*, together with the higher expression of the nuclear receptor *PPARG*, a master regulator of adipogenesis and obesity^28^, may explain the higher number of over-expressed genes observed in females related to lipogenic pathways and fat deposition. Furthermore, it is relevant to highlight the over-expression in females of other nuclear receptors such as *LXRA* and *RXRG*. *LXR*s activation in human skeletal cells promote increased uptake, synthesis, utilization and storage of lipid^29^. Finally, the *IGF2* gene, which has been involved in muscle growth and fat deposition^22^, showed also a higher expression in females.

**Figure 2:**
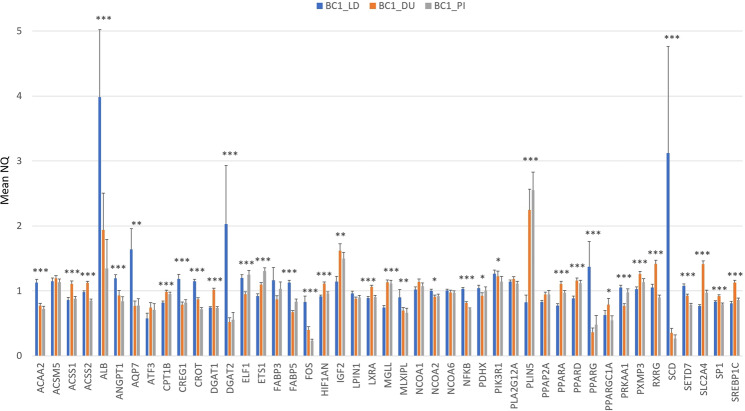
Comparison between the three experimental backcrosses in the mRNA expression levels of 45 lipid-related genes. Data represents means ± standard error of the mean (SEM). Significant differences are labelled as *p-value ≤ 0.05, **p-value ≤ 0.01 and ***p-value ≤ 0.001.

A breed effect on the expression of genes involved in energy balance and lipogenesis was reported in a comparison between Iberian and Duroc pigs^30^. In our study, a significant backcross effect (*p*-value≤ 0.05) on gene-expression levels was detected in 37 out of the 45 genes analysed: *ACAA2*, *ACSS1*, *ACSS2*, *ALB*, *ANGPT1*, *AQP7*, *MLXIPL*, *CPT1B*, *CREG1*, *CROT*, *DGAT1*, *DGAT2*, *ELF1*, *ETS1*, *FABP5*, *FOS*, *HIF1AN*, *IGF2*, *LXRA*, *MGLL*, *NCOA2*, *NFKB*, *PDHX*, *PIK3R1*, *PLIN5*, *PPARA*, *PPARD*, *PPARG*, *PPARGC1A*, *PRKAA1*, *PXMP3*, *RXRG*, *SCD*, *SETD7*, *SLC2A4*, *SP1* and *SREBP1C* (Fig. 2). Overall, 18 and 16 out of 45 genes were over-expressed in BC1_LD and BC1_DU respectively, and are involved in a wide range of functions. In summary, genes more related to lipogenic pathways were more expressed in BC1_LD (*DGAT2*, *PPARG*, *NCOA2*, *SCD* and *PRKAA1*) whereas genes related to lipolytic pathways were higher expressed in BC1_DU (*ACSS1*, *ACSS2*, *CPT1B*, *PPARA* and *PPARGC1A*). Finally, 3 out of 45 genes were over-expressed in BC1_PI and were mainly related to transcriptional regulation and control (*ELF1*, *ETS1* and *PLIN5*).

The high number of lipid-related genes differentially expressed between breeds may be caused by different selection according to breed in pigs, such as in fat content. In our animal material, the Iberian pig is characterized by a high content of SFA, MUFA and IMF, conferring a good meat quality. On the other hand, Landrace pigs are considered very lean with less IMF and high PUFA content, Duroc pigs are more fat with an improved carcass meat quality and Pietrain animals present a good carcass conformation. Moreover, other studies reported differences in the expression of genes involved in specific metabolic pathways depending on FA traits^16,31^.

These differences in gene expression pattern may indicate differences in gene-expression regulatory mechanisms among breeds, which are described below.

Altogether these results indicated an effect of sex and breed on gene expression, therefore they were considered in association studies and included as co-factors in the model.

### Gene expression correlations

**Figure 3:**
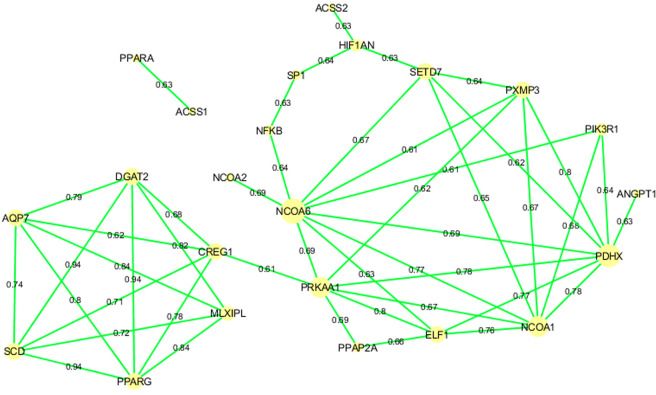
Gene co-expression network in 3BCs using the PCIT algorithm. After filtering by significance and r ≥ 0.6, 23 from the 45 initial genes are shown. Node size represents the degree of a node.

In order to identify co-expression patterns in the selected genes analysed in our study, a co-expression network using PCIT^32^ algorithm was performed with the expression data of 3BCs animals (Fig. 3).

Two groups of co-expressed genes were identified by PCIT. It is particularly interesting to mention the strongest correlations found for the lipogenic genes *SCD*, *PPARG*, and *DGAT2* in the first group and which were previously identified in the BC1_LD study^17^. *CREG1* and *PRKAA1* were identified linking both groups of co-expressed genes. Remarkably, among this second group of co-expressed genes strong correlations for *ELF1*, *NCOA1*, *NCOA6*, *PDHX*, *PRKAA1*, *PXMP3* and *SETD7* were identified and the highest node degree corresponded to *NCOA6* and *PDHX*.

Hence, genes involved in lipogenesis are highly correlated in porcine muscle, suggesting a coordinated regulation of its expression. In previous studies of our group, two groups of extreme animals for intramuscular fatty acid composition were analysed by RNA-Seq, showing an increase in fatty acid and glucose uptake, and also an increase in the lipogenesis pathway in the muscle of pigs with higher levels of MUFA and SFA content^16^. Moreover Puig-Oliveras *et al*. (2016), showed a positive correlation between lipogenic genes and palmitoleic and octadecenoic fatty acids, and a positive correlation between lipolytic genes and PUFAs, specifically with the linoleic fatty acid in the BC1_LD animals^17^.

### Genome-wide association studies for gene expression and eQTL identification

**Figure 4:**
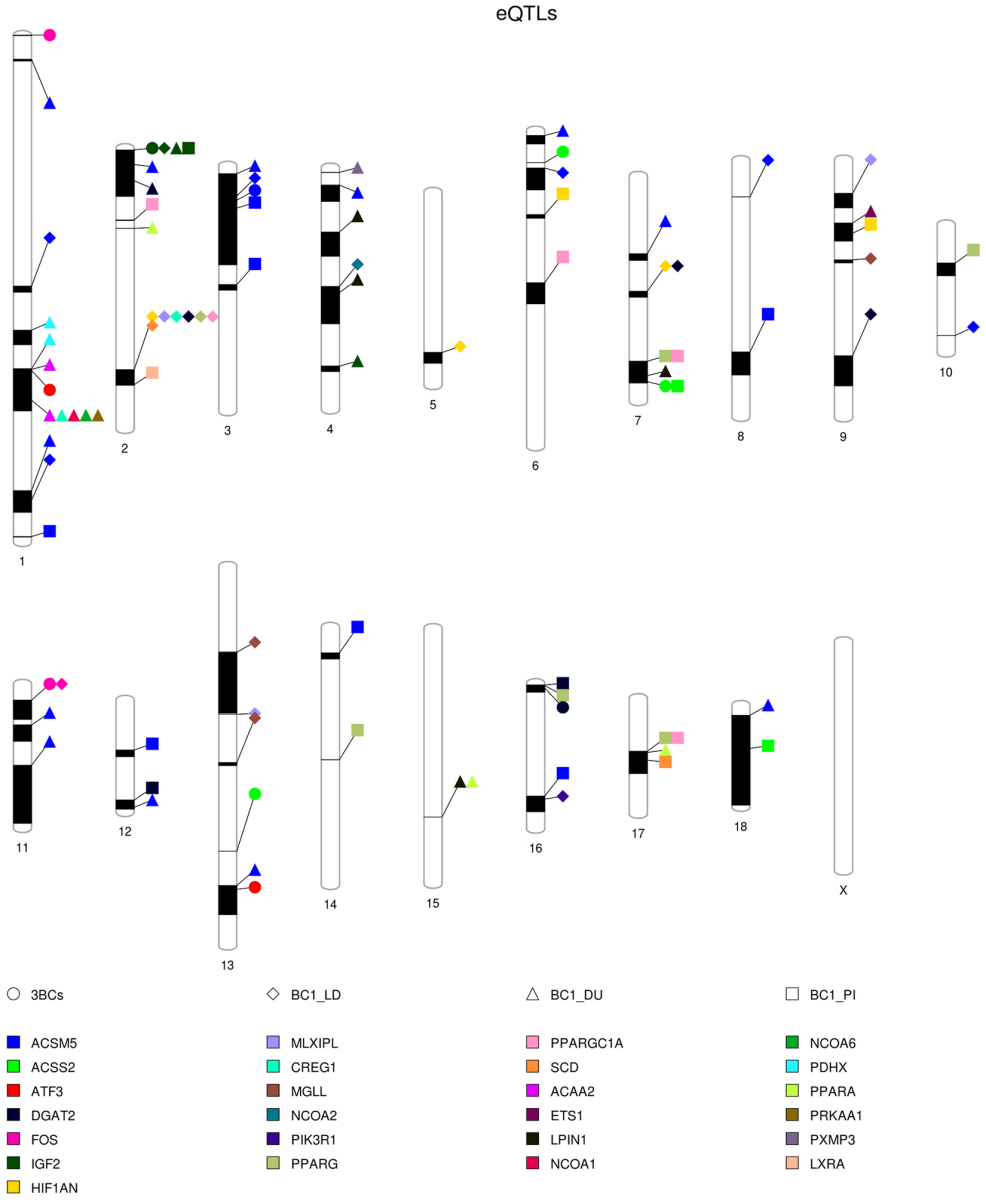
PhenoGram plot representing associated gene expression regions along pig chromosomes in the 3BCs study and in each backcross individually. The shape indicates the backcross or the 3BCs altogether and the colour indicates the gene name as it is indicated in the legend.

An eGWAS was performed with the muscle gene expression values and the genotypes of 38,426 single nucleotide polymorphisms (SNPs) distributed along all chromosomes in 355 3BCs animals. The eGWAS identified 186 expression-SNPs (eSNPs) located in 10 *Sus scrofa* chromosomes (SSC) regions of SSC1, SSC2, SSC3, SSC6, SSC7, SSC11, SSC13 and SSC16 and associated with the expression of *ACSM5*, *ACSS2*, *ATF3*, *DGAT2*, *FOS* and *IGF2* (FDR < 0.05) genes (Supplementary Table S1). Ten eQTLs showed *trans*-regulatory effects on gene expression and two of them, *IGF2* and *ACSM5*, were also classified as *cis*-acting, suggesting that there is a mutation in the same gene or in a proximal genomic region affecting its expression (Table 1). Both *cis* and *trans-*eQTLs were represented in Fig. 4.

**Table 1:** Significant eQTLs for the 45-muscle gene expression study in 3BCs animals.

Interval	Gene	Chr.	Start Position (bp)	End Position (bp)	Size (bp)	SNPs	Type of eQTL	Candidate genes
1	*ACSM5*	3	18,557,492	53,699,303	35,141,811	58	*cis/trans*	*ACSM5* and *IL4R*
2	*ACSS2*	6	17,315,441	17,502,570	187,129	2	*trans*	
3	*ACSS2*	7	111,283,606	112,227,872	944,266	8	*trans*	
4	*ACSS2*	13	156,576,634	156,644,710	68,076	2	*trans*	
5	*ATF3*	1	181,624,438	181,702,614	78,176	3	*trans*	
6	*ATF3*	13	177,313,258	177,546,824	233,566	2	*trans*	
7	*DGAT2*	16	2,764,727	2,779,416	14,689	2	*trans*	
8	*FOS*	1	0	493,510	493,510	2	*trans*	
9	*FOS*	11	8,855,571	19,677,423	10,821,852	3	*trans*	*RB1* and *FOXO1*
10	*IGF2*	2	1,000,000	25,964,207	24,964,207	104	*cis/trans*	*IGF2, SF1* and *NR1H3*

Start and end positions refer to the eQTL interval and are based on Sscrofa 11.1 assembly. Gene annotation was performed considering one additional Mb at the start and at the end of the eQTL interval. SNPs column indicates the number of SNPs within the eQTL interval. For the *cis*-eQTLs regions only the analyzed gene was annotated as positional candidate gene.

#### Cis-eQTLs

**Figure 5:**
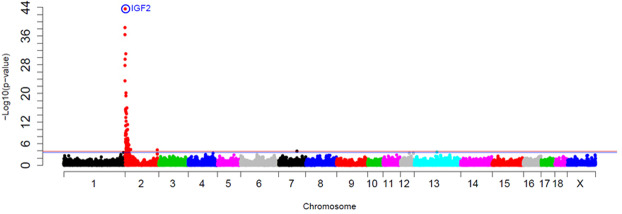
GWAS plot of muscle *IGF2* gene expression in the 3BCs study. Chromosome positions in Mb based on Sscrofa11.1 assembly of the pig genome are represented in the X-axis and the –log10 (*p*-value) is on the Y-axis. Horizontal lines represent the genome-wide significance level (FDR-based *q*-value <0.1 corresponds to blue line and FDR-based *q*-value <0.05 to red line). The *IGF2*:g.3072 G > A polymorphism is circled and labelled as IGF2 in colour blue.

For the *IGF2 cis*-eQTL region, the *IGF2g.3072 G* > *A* SNP was the most significantly associated polymorphism (*p*-value = 3.24 × 10^−44^) and explained the 70% of the muscle *IGF2* expression variance, approximately (Fig. 5).

The *IGF2:g.3072 G* > *A* substitution has been identified as the causal mutation of an imprinted QTL for muscle growth, fat deposition and heart size^22^ and it is maternally imprinted in most animal tissues^33^. The *IGF2g.3072 G* > *A* mutation is located in a well-conserved CpG island, which is hypomethylated and abrogates the binding site for an *IGF2* transcriptional repressor called ZBDE6, leading to a three-fold up-regulation of the *IGF2* expression in pig skeletal muscle^22,34^.

**Figure 6:**
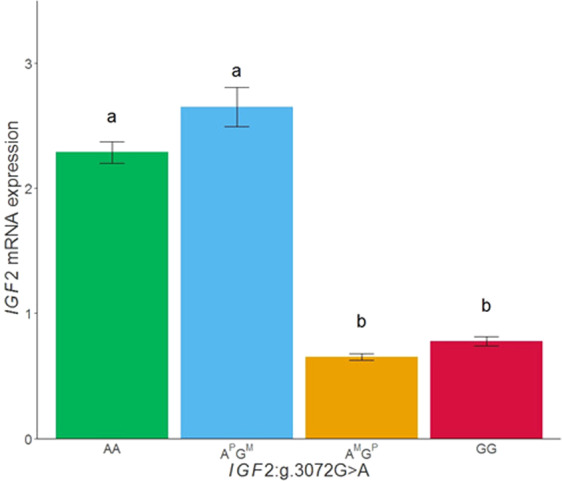
Plot of mRNA expression values (NQ) of *IGF2* in muscle tissue according to the *IGF2*:g.3072 G > A genotype. A^P^G^M^ indicates a paternally inherited A allele and maternal inherited *G* allele, on the contrary, A^M^G^P^ represents a maternal inherited A allele and paternal inherited G allele. Data represents means ± standard error of mean (SEM). Values with different superscript letters (a, b) indicate significant differences between groups (*p*-value < 0.05).

An imprinting model was tested for muscle gene expression in 327 animals in which the paternal allele was deduced from progenitor’s genotypes (Fig. 6). Animals with the paternally-inherited A allele (A^P^) of the *IGF2:g.3072 G* > *A* polymorphism showed the highest *IGF2* gene expression in muscle (AA: NQ mean = 2.29, n = 130 and A^P^G^M^: NQ mean = 2.65, n = 26) compared to animals with paternally-inherited G allele (A^M^G^P^: NQ mean = 0.65, n = 122 and GG: NQ mean = 0.78, n = 76).

Therefore, the *IGF2*:g.3072 G > A SNP genotype and the imprinting model explained the differences observed in *IGF2* gene expression in muscle, being the *IGF2* genetic variant the major regulator of gene expression in muscle in different genetic backgrounds (see below specific data for each backcross).

A previous study of our group reported that *IGF2* polymorphism was also the most significant associated SNP with *IGF2* mRNA expression in adipose tissue, but it explained only 25% of the phenotypic variance compared to the 70% explained in muscle tissue, suggesting that other genetic variants, potentially *trans*-regulation as reported in the current study, may affect the gene expression in adipose tissue. Nevertheless, the *IGF2* gene expression followed a maternal imprinting model in both tissues^35^.

The *ACSM5* gene, target of the other *cis*-eQTL region identified, is involved in pathways such as conjugation of carboxylic acids and FA beta-oxidation. A SSC3 *cis*-eQTL was reported in a previous study of our group analysing the *ACSM5* expression in BC1_LD population^17^. The *ACSM5* proximal promoter region was amplified and sequenced in ten BC1_LD animals and subsequently three polymorphisms were found. The most proximal 5’ mutation, *rs331702081* (hereinafter known as *ACSM5.P*) was the most significantly associated SNP with the *ACSM5* gene expression in the BC1_LD population^17^. Thus, in the current study the *ACSM5.P* was genotyped in the BC1_DU and BC1_PI populations.

**Figure 7:**
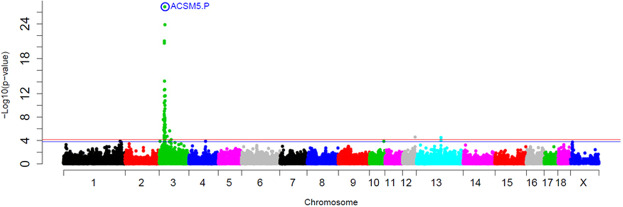
GWAS plot of muscle *ACSM5* gene expression in the 3BCs study. Chromosome positions in Mb based on Sscrofa11.1 assembly of the pig genome are represented in the X-axis and the –log10 (*p*-value) is on the Y-axis. Horizontal lines represent the genome-wide significance level (FDR-based *q*-value < 0.1 corresponds to blue line and FDR-based *q*-value < 0.05 to red line).

In the eGWAS with all three backcrosses the *ACSM5.P* SNP presented the strongest association with muscle *ACSM5* gene expression (*p*-value = 1.39 × 10^−27^) (Fig. 7). The polymorphism located in the promoter region explained approximately the 40% of the phenotypic variance, suggesting the presence of additional genetic factors regulating its gene expression (see below specific data for each backcross). Further analysis should be done to understand the transcriptional regulation of *ACSM5* gene.

In a previous study of our group the *ACSM5.P* mutation has been also described as the most significantly associated SNP with *ACSM5* gene expression in backfat adipose tissue of the BC1_LD population. Nonetheless, the correlation between the *ACSM5* gene expression in backfat and muscle was 0.60, suggesting that the gene expression in both tissues could be regulated by different genetic variants. In addition, two transcription factors (*ARNT* and *STAT6*) that bind only with the A allele is present^19^ were identified. Hence, genetic variation on the promoter region of *ACSM5* could be a key regulator of the *ACSM5* gene expression, at least in muscle and adipose tissues.

#### Trans-eQTLs

A total of 783 genes were located in the 10 *trans*-eQTL genomic regions identified in our study. Among them, we identified potential lipid metabolism regulatory genes in three regions (Table 1: interval 1, 9 and 10). The *ACSM5* eGWAS revealed a *trans*-eQTL located in the 18.5 Mb – 53.6 Mb region of SSC3, where the Interleukin 4 Receptor (*ILR4*) gene was mapped. Polymorphisms in *ILR4* have been associated with high density lipoprotein-cholesterol levels, suggesting the possible role of *IL4R* gene in lipid metabolism in humans^36^. The FBJ Murine Osteosarcoma Viral Oncogene Homolog (*FOS*) eGWAS revealed a *trans*-eQTL in the 8.9 Mb − 19.7 Mb region of SSC11, where a gene involved in lipid metabolism was mapped: Forkhead Box O1 (*FOXO1*). From the *FOXO* transcription factor family, *FOXO1* is the isoform with the highest expression in muscle and has been proposed as a regulator of energy metabolism and the insulin signalling pathway^37^. It is also involved in muscle differentiation and can interact with other transcription factors such as *PPARG* and *HNF4A* to regulate insulin gene expression and IMF accumulation^38^. Moreover, *FOXO1* was found to regulate *FOS* gene expression in skeletal muscle, increasing their levels during cancer cachexia in humans^39^. Retinoblastoma 1 (*RB1)* gene was also a transcription factor mapped in this region and is involved in gene expression control. *RB1* plays an important role in cell cycle and cell differentiation and is also considered as a key regulator during adipogenesis. However, it is highly expressed in muscle tissue probably due to its role in muscle differentiation^40^. In humans, *RB1* was found co-expressed with *FOS* gene and is involved in proliferation and apoptosis in myosarcoma^41^. A prediction of a functional integration network was done by GeneMANIA, showing a gene co-expression between *FOS* and *FOXO1*, a predicted functional gene relationship between *FOS* and *RB1*, and *FOXO1* with *PPARG* and *HNF4*, protein-protein interactions among *FOXO1* and *RB1* and finally a *FOS*, *PPARG* and *RB1* gene pathway.

The Splicing Factor 1 (*SF1*) gene was mapped in the *IGF2 trans*-eQTL region located on SSC2 (Table 1) and it was previously described as a candidate gene for *IGF2* regulation in adipose tissue^35^. A member of the *LXR* nuclear receptor family named nuclear receptor subfamily 1 group H member 3 (*NR1H3*) was also mapped in this *trans*-eQTL region and chosen as a possible candidate gene due to its involvement in the deposition of lipids in pigs, which may affect lean muscle fat content^42^.

The rest of the *trans*-eQTL regions were identified for *ACSS2* (SSC6, SSC7 and SSC13), *ATF3* (SSC1 and SSC13), *DGAT2* (SSC16) and *FOS* (SSC1). However, no candidate regulator genes could be identified in these genomic regions. This may be explained by the small intervals size, the lack of gene information in the pig assembly or the presence of other regulators such as enhancers, miRNAs and long-non-coding RNAs among others.

### eGWAS analysis for each backcross independently

Expression-GWAS studies were also performed for each backcross independently and 420, 420 and 224 associated eSNPs were identified in the BC1_LD, BC1_DU and BC1_PI animals, respectively (Supplementary Table S2). A total of 26 eQTLs were found in BC1_LD located on SSC1-SSC11, SSC13 and SSC16. Of these, two *trans*-eQTLs were previously reported in the study with the same BC1_LD animals using the previous *Sscrofa* 10.2 assembly^17^ (see below). In BC1_DU, 32 eQTLs were detected on SSC1-SSC4, SSC6-SSC7, SSC9, SSC11-SSC13, SSC15, SSC17, and SSC18, and the 25 eQTLs found in BC1_PI were located on SSC1-SSC3, SSC6-SSC10, SSC12, SSC14 and SSC16-SSC18, and are represented in Fig. 4 (Supplementary Table S3).

#### Cis-eQTLs

The *cis*-eQTL regions of *ACSM5* and *IGF2* genes, on SSC33 and SSC2 respectively, appeared segregating in all three backcrosses, which suggest that the Iberian boars and the three founder maternal breeds have different allelic frequencies for the polymorphisms regulating *in cis* the expression of these genes.

The *ACSM5.P* polymorphism was segregating at low frequencies, being the *ACSM5.P A* allele frequency of 0.22 in BC1_LD, 0.09 in BC1_DU and 0.10 in BC1_PI. In the BC1_LD the *ACSM5.P* SNP was the most significant polymorphism associated with the differences in the mRNA level of *ACSM5* and explained around the 58% of the phenotypic variance. As stated before, this result agrees with the previous study performed with the same BC1_LD but in which the *Sus Scrofa* 10.2 genome assembly was used^17^. In BC1_PI, *rs81475068*, *rs81278505* and *ACSM5.P* polymorphisms were located on SSC3 and spanning 0.17 Mb (2.39–2.56 Mb) and were the most significant associated SNPs with *ACSM5* gene expression (*p*-value = 7.32 × 10^−09^), explaining approximately the 28% of the phenotypic variance. Contrarily, in BC1_DU *rs81327383* was the most significantly associated SNPs (*p*-value = 2.02 × 10^−12^) with *ACSM5* mRNA expression although the *ACSM5.P* polymorphism was also significant (*p*-value = 3.44 × 10^−09^) and explained a 26% of the gene expression variance. Hence, the lack of allele segregation or the presence of other proximal genetic variants could be involved in these gene expression changes.

In a previous work performed only in BC1_LD animals, the *cis*-eQTL for the muscle *IGF2* gene expression was identified, but the *IGF2*:*g.3072 G* > *A* polymorphism was not the most significant associated SNP, which was *rs335265872* (called *DIAS0000846* and located at 6.20 Mb on SSC2 in the *Sus Scrofa* 10.2 genome assembly)^17^. In the present work, the most significantly associated SNP in BC1_LD (*p*-value = 1.45 × 10^−15^) was r*s81322199* located at 3.68 Mb on SSC2 and explaining the 42% of the phenotypic variance. In addition, the *IGF2g.3072 G* > *A* polymorphism was significantly associated (*p*-value = 3.03 × 10^−07^) and explained the 22% of the *IGF2* mRNA variation. This result may be explained by the low number of homozygous AA animals, being 0.2 the allele frequency of the *IGF2:g.3072 A* allele. On the other hand, the *IGF2g.3072 G* > *A* polymorphism was the most significantly associated SNP with *IGF2* gene expression in BC1_DU (allele frequency of 0.29) and BC1_PI (allele frequency of 0.23), explaining in both cases a high proportion of the gene expression variance, 58% and 92% respectively. In BC1_DU other genomic regions seem to be also associated with the *IGF2* gene expression differences, as the eQTL located in the 107.4–110.8 Mb genomic region of SSC4.

Two more *cis*-eQTLs were identified only in the BC1_LD population for *MGLL* and *NCOA2* gene expression. The *MGLL* eQTL was previously described in the same backcross^17^. The SSC4 *cis*-eQTL for *NCOA2* gene expression presented four significant associated SNPs, being the *rs80803396* the SNP showing the strongest signal (*p*-value = 2.32 × 10^–06^).

#### Hotspots identified in trans-eQTLs regions

All the *trans*-eQTLs intervals, eSNPs and annotated candidate genes are shown in the supplementary table S1, but only eQTL hotspots are discussed in detail (Supplementary Table S4). In BC1_DU, new *trans*-eQTLs were identified for *ACAA2* (SSC1), *ACSM5* (SSC1, SSC2, SSC4, SSC6, SSC7, SSC11, SSC12, SSC13, and SSC18), *CREG1* (SSC1), *DGAT2* (SSC2), *ETS1* (SSC9), I*GF2* (SSC4), *LPIN1* (SSC4, SSC7, and SSC15), *NCOA1* (SSC1), *NCOA6* (SSC1), *PDHX* (SSC1), *PPARA* (SSC2, SSC15, and SSC17), *PRKAA1* (SSC1), and *PXMP3* (SSC4) genes in comparison to the 3BCs study. In BC1_LD additional *trans*-eQTLs were found for *ACSM5* (SSC1, SSC6, SSC8, and SSC10), *MLXIPL* (SSC2, SSC9, and SSC13), *CREG1* (SSC2), *DGAT2* (SSC2, SSC7, and SSC9), *HIF1AN* (SSC2, SSC5, and SSC7), *MGLL* (SSC9 and SSC13), *PIK3R1* (SSC16), *PPARG* (SSC2), *PPARGC1A* (SSC2), and *SCD* (SSC2) genes in comparison to the 3BCs study. Two of the *trans-*eQTLs identified in the BC1_LD (*FOS* at SSC11 and *MGLL* at SSC13) were previously reported in the same BC1_LD animals with the *Sus Scrofa* 10.2 genome assembly. Finally, new *trans*-eQTLs in BC1_PI were detected for *ACSM5* (SSC1, SSC8, SSC12, SSC14, and SSC16), *ACSS2* (SSC18), *DGAT2* (SSC12), *HIF1AN* (SSC6 and SSC9), *LXRA* (SSC2), *PPARG* (SSC7, SSC10, SSC14, SSC16, and SSC17), *PPARGC1A* (SSC2, SSC6, SSC7, and SSC17), and *SCD* (SSC17) genes (Fig. 4) in comparison to the 3BCs study.

We only observed three common *trans*-eQTL regions in the 3BCs study, suggesting the presence of different regulatory mechanisms or frequencies according to breed. Overall, the *trans*-eQTL regions manifested that the expression of the genes related to lipid metabolism is regulated in a complex way.

In addition, six hotspots regions, two in each backcross, regulating the expression of several genes were detected.

**Figure 8:**
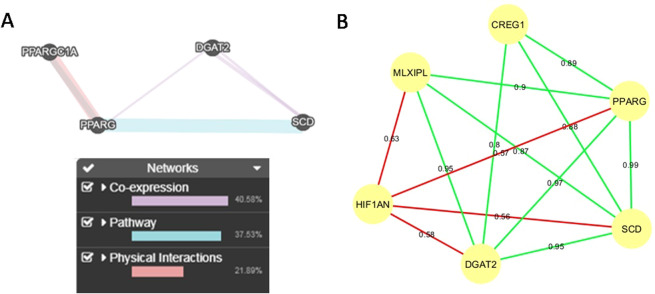
(**A**) GeneMANIA analysis between SSC2 hotspot genes. (**B**) Co-expression network using the PCIT algorithm within the genes associated with the BC1_LD *trans*-eQTL hotspot region on SSC2. Red and green lines indicate negative and positive correlations respectively.

In BC1_LD animals a *trans*-eQTL hotspot located on SSC2 and spanning 8.7 Mb (119.9–128.7 Mb) was associated with the expression of seven genes: *HIF1AN*, *CREG1*, *MLXIPL*, *DGAT2*, *PPARG*, *PPARGC1A*, and *SCD*. After gene annotation of this region no candidate *trans*-acting regulators modulating the expression of genes on the SSC2 hotspot were found. However, the transcription factor 7 (*TCF7*) gene was annotated in the *CREG1* eQTL region because it was six Mb longer (119.9–136.2 Mb) than the others. *TCF7* and its family member transcription factor 7 like 2 (*TCF7L2*) have been associated with diabetes in humans^43^. In addition, *TCF7L2* has been described as an indirect regulator of PPARD during adipogenesis^44^. In addition, to evaluate potential functional interactions and the co-expression pattern of genes on the SSC2 hotspot, GeneMANIA and PCIT co-expression network analysis were done (Fig. 6). Interactions between *DGAT2*, *PPARG*, *PPARGC1A* and *SCD* were found with GeneMANIA. (Fig. 8A). In general, meaningful gene-gene interactions were shown by PCIT (Fig. 8B), reinforcing the presence of a common regulatory factor modulating the expression of SSC2 hotspot genes. However, lower correlations were observed for the *CREG1* gene, suggesting the presence of an independent regulatory factor modulating its expression. This result is in accordance with the proposal of the *TCF7* as a candidate gene of this region, although further validations are needed. Furthermore, *HIF1AN* presented negative and moderate correlations with *DGAT2*, *MLXIPL*, *PPARG*, and *SCD*, suggesting an opposite regulatory effect for this gene. *HIF1AN* is involved in fatty acid β-oxidation^45–47^, while *DGAT2*, *PPARG*, *MLXIPL* and *SCD* are related to *de novo* lipogenesis, triacylglycerol synthesis and adipogenesis^16,48–50^.

The strong correlation for *SCD*, *PPARG* and *DGAT2* identified in the gene co-expression network in 3BCs, and with *MLXIPL* and *CREG1* have been found associated altogether with the *trans*-eQTL hotspot on SSC2 in the BC1_LD study but not in the other two backcrosses (BC1_DU and BC1_PI).

The region spanning 3.5 Mb on SSC7 (62.4–65.9 Mb) presented significant associations with the *HIF1AN* and *DGAT2* gene expression. The nuclear factor of kappa light polypeptide gene enhancer in B cells inhibitor alpha (*NFKBIA*) gene was mapped in this region. It is a transcription factor involved in immune response, but also plays a direct role in adipogenesis and fat accumulation^51,52^. *NFKBIA* was found differentially expressed in different development stages and muscles between Iberian and Iberian x Duroc pigs, suggesting that it is a molecular regulator of metabolism^38^. An experimental interaction between *HIF1AN* and *NFKBIA* was identified by GeneMANIA and String programs, but no information about *DGAT2* interactions was found, so further validation will be needed to corroborate our results. Hence, we can suggest that *NFKBIA* is involved in muscle lipid metabolism, being an interesting candidate gene to explain the differences in the expression of two genes associated with the SSC7 hotspot in BC1_LD animals. None of these hotspots were identified in the previous study in which the same BC1_LD individuals were analyzed^17^. Taking all the eQTL results from BC1_LD together, discrepancies between our results and the work of Puig-Oliveras *et al*. (2016)^17^ may be explained by the different genome assemblies used between both works, being *Sus Scrofa* 10.2 genome assembly in the previous work and *Sus Scrofa* 11.1 in the present one.

**Figure 9:**
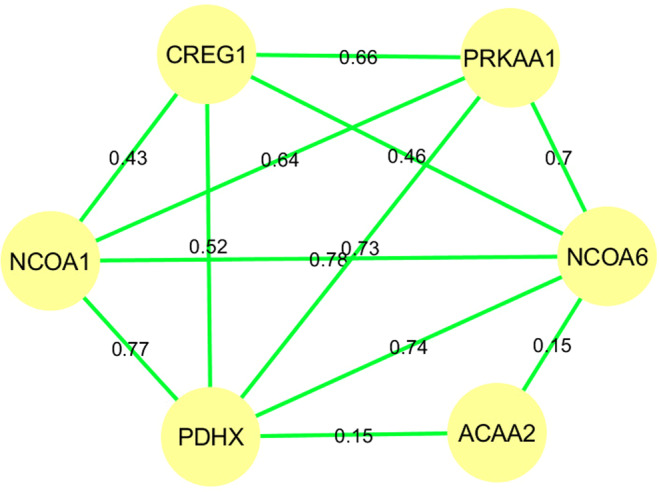
Co-expression network for genes associated with the BC1_DU *trans*-eQTL hotspot on SSC1 using the PCIT algorithm.

In the BC1_DU animals study two *trans*-eQTL hotspot regions were found on SSC1, spanning 6 Mb (180.6–203.6 Mb), and on SSC15, spanning 0.3 Mb (103.7–104 Mb). The SSC1 region showed significant associations with the expression of the *ACAA2*, *CREG1*, *NCOA1*, *NCOA6*, *PDHX* and *PRKAA1* genes. The perilipin 2 (*PLIN2*) gene was mapped in this region but was only annotated as a candidate gene for *ACAA2*, *NCOA1*, *NCOA6* and *PDHX*. *PLIN2* was reported to be involved in the uptake and storage of FAs in human skeletal muscle^53^. Studies in pigs described that a higher *PLIN2* gene expression was associated with a higher IMF content in muscle^54,55^. In order to deep in the study of the genes regulated by the same eQTL on SSC1, the PCIT algorithm was used to build a co-expression network. Moderate to high positive correlations, from 0.15 to 0.78, were observed among the genes regulated by the same eQTL (Fig. 9). Lower correlations were observed for *ACAA2*, a gene encoding an enzyme that catalyzes the last step in mitochondrial fatty acid β-oxidation^56^, suggesting the presence of another genetic factor regulating its expression. In addition, moderate correlations were found for the rest of the hotspot genes, mainly related to transcriptional regulation and control.

Notably, the second group of genes identified in the gene co-expression network in 3BCs, which showed strong correlations for *PRKAA1*, *PDHX*, *NCOA1* and *NCOA2* among others, coincides with the previously observed SSC1 *trans*-eQTL hotspot in BC1_DU study, but not in the other two backcrosses (BC1_LD and BC1_PI).

*LPIN1* and *PPARA* genes were significantly associated with the SSC15 hotspot region and showed a moderate correlation value (*LPIN1*-*PPARA*, r = 0.59 *p*-value = 4.97 × 10^−13^). In this region was mapped a key mitochondrial enzyme for fatty acid oxidation, *AOX1* gene. It has been reported to be associated with FA oxidation in mice adipocytes^57^ and meat quality traits and with muscle development in cattle^58^.

Regarding BC1_PI population, two *trans*-eQTL hotspots regions on SSC7 and SSC17 were observed. The first region, spanning 8.1 Mb on SSC7 (100.1–108.2 Mb), showed a significant association with *PPARG* and *PPARGC1A* gene expression. *DIO2* gene was mapped in the SSC7 *trans*-eQTL region as a potential candidate gene for lipid metabolism. It has been selected as a muscle candidate gene in an obesity resistance study since it presented differences between lean and fat mouse lines^59^. *DIO2* converts prohormone thyroxine (T4) to the active hormone triiodothyronine (T3), which binds to tyroid hormone receptors (TR). TR heterodimerize with RXR and can compete with PPAR for that binding site affecting gene control and regulation^60^. Hence, *DIO2* may be an indirect regulator of SSC7 hotspot genes.

The second region located on SSC17 and spanning 12.6 Mb (29.2–41.8 Mb), presented a significant association with *PPARG*, *PPARGC1A* and *SCD*. Three genes were mapped for the SSC17 hotspot: *RBL1*, *FOXA2* and *E2F1*. *RBL1* gene has been associated with the whole body fat metabolism and determines the oxidative state of muscle in mice^61^. *FOXA2* has been described as a transcription factor of several genes involved in the insulin pathway in liver^62^, but no studies in muscle tissue were found. It was reported that *E2F1* is required for *in vivo* skeletal muscle regeneration in mouse^63^ and showed high gene expression levels in Pietrain pigs with high muscle content^64^. Interactions were found between the genes associated with the hotspot (*PPARG*, *PPARGC1A* and *SCD)* and between the *E2F1*, *RBL1* and *PPARG* genes using GeneMANIA and String*. RBL1* and *E2F1* were selected as promising candidate genes for lipid metabolism in pigs, but further validations are needed to assess the effect of *FOXA2* in muscle tissue.

## Conclusions

In the present study, gene expression of candidate genes for fatty acid composition in muscle showed sex-dimorphism and breed effects, and gene co-expression in different lipid metabolism pathways was identified. The eGWAS revealed two *cis*-eQTL and ten *trans*-eQTL regions associated with the muscle expression of *ACSM5*, *ACSS2*, *ATF3*, *DGAT2*, *FOS* and *IGF2* genes. Both *IGF2*:g.3072 G > A and *ACSM5.P* polymorphisms were the most significant SNPs associated with the *IGF2* and *ACSM5* gene expression levels, respectively, in different pig genetic types. Two *trans*-eQTL hotspot regions per backcross regulating the expression of up to seven genes were identified, and a list of candidate genes involved in the regulation of these eQTL regions was reported. Overall, our results increase the knowledge of the genetic basis of gene expression regulation in muscle lipid metabolism.

## Material and Methods

### Animal material

A total of 355 pigs (called 3BCs) belonging to three different experimental backcrosses were studied: 114 BC1_LD (25% Iberian and 75% Landrace), 122 BC1_DU (25% Iberian and 75% Duroc), and 119 BC1_PI (25% Iberian and 75% Pietrain)^35^. All animals were maintained under the same intensive conditions and fed *ad libitum* with a cereal-based commercial diet and slaughtered in a commercial abattoir following institutional and national guidelines for the Good Experimental Practices and approved by the Ethical Committee of the Institution (IRTA – Institut de Recerca i Tecnologia Agroalimentàries). In addition, animal care and procedures were carried out according to the Spanish Policy for Animal Protection RD1201/05 and the European Union Directive 86/609 about the protection of animals used in experimentation.

LD samples were collected at slaughterhouse in liquid nitrogen and stored at −80 °C until analysis. Genomic DNA was extracted from diaphragm tissue using the phenol-chloroform method^65^.

### Genotyping

Animals from BC1_LD and BC1_PI were genotyped using the Porcine SNP60K BeadChip (Illumina, San Diego, USA) and BC1_DU animals were genotyped using the GeneChip Porcine Genome Array (Affymetrix). Only SNPs that mapped against the Sscrofa11.1 assembly and were common to both arrays were selected^35^. Markers that showed a minor allele frequency (MAF) lower than 5% and SNPs with more than 5% of missing genotypes were removed with Plink software^66^. Moreover, based on the information in the prior BC1_LD study^17^, two additional SNPs were genotyped: *ACSM5* (*rs331702081*) and *IGF2* (*IGF2*:g.3072 G > A), in the BC1_DU and BC1_PI populations, following the previously described protocols^17,22^. Finally, a total of 38.426 SNPs distributed along all chromosomes, including *rs331702081* and *IGF2*:g.3072 G > A polymorphisms, were used for association studies.

### Gene expression

Total RNA was obtained from the LD muscle of 355 animals using the RiboPure kit (Ambion), following the fabricator’s instructions. RNA quantification and purity was performed with a NanoDrop ND-1000 spectrophotometer (NanoDrop products) and RNA integrity was assessed by Agilent Bioanalyzer-2100 (Agilent Technologies). The RNA was reverse-transcribed into cDNA using the High-Capacity cDNA Reverse Transcription kit (Applied Biosystems), following the manufacturer’s recommendations.

Gene expression was analyzed in 48 genes, of which 45 were target genes and *ACTB*, *HPRT1* and *TBP* were the candidate reference genes, by quantitative real time-PCR (qPCR). Selection of target genes related to lipid metabolism as well as primer design details and sequences was described in Puig-Oliveras *et al*. (2016)^17^. Gene expression quantification was performed in a 48.48 Microfluidic Dynamic Array IFC Chip (Fluidigm) in a BioMark System succeeding a previously described protocol^67^. Gene expression data was picked up using Fluidigm Real-Time PCR analysis software 3.0.2 (Fluidigm) and analyses were done with DAG Expression software 1.0.4.11^68^, applying the relative standard method curve. *ACTB* and *TBP* were used as the most stable reference genes, while *HPRT1* was discarded, in order to normalize the expression levels of target genes. The normalized quantity (NQ)^68^ values of each sample and assay were used to compare the expression data among animals. Normalization of data was checked through Shapiro-Wilk test in R^69^, and log_2_ transformation of the NQ value was applied if necessary. Sex and breed effects were tested by using a linear model (lm) in R^69^.

### Genome-wide association analysis for gene expression

To carry out the genomic association studies between 45 gene expression values and common SNPs genotypes (eGWAS), a previously reported^35^ linear mixed model using the GEMMA software^70,71^ was applied.

### Gene annotation

Significant associated SNPs were mapped in the Sscrofa11.1 assembly and were annotated with the Ensembl Genes 91 Database using VEP software^72^. BioMart software^73^ was used to annotate genomic eQTL intervals considering ±1 Mb around the candidate chromosomal regions. In the three studied BCs study only eQTL intervals containing 2 or more SNPs were annotated, whereas in the individual backcross GWAS annotation was done for eQTL intervals containing 3 or more SNPs.

The identified SNPs were classified depending on their location, as *cis* if the SNPs were located within 1 Mb of the analyzed gene and as *trans* if the SNPs were located elsewhere in the genome. The number of significant SNPs belonging to the same interval was considered among associated SNPs less than 10 Mb apart.

### Co-expression and functional analysis

The PCIT algorithm was used to calculate weighted gene co-expression networks, through the implementation of first-order partial correlations coefficients combined with information theory approach, in order to identify principal interactions between genes^32,74^. Only the significant interactions between genes were considered for further steps. Networks were represented with CentiScaPe Cytoscape plug-in^75^.

Ingenuity Pathway Analysis software (IPA; Ingenuity Systems) and the *Core Analysis* function was used to perform functional analysis of genes mapped in the different intervals and for data interpretation in the context of biological processes, pathways and networks. In addition, the iRegulon v1.3. Cytoscape plug-in^76^ was used to identify transcription factor (TF) binding sites in silico. ClueGO plug-in^77^ was used to integrate and cluster the genes regarding their Gene Ontology and KEGG pathway. Finally, GeneMANIA^78^ and String^79^ were used to evaluate the functional interaction and networks among genes proteins, respectively.

## Supplementary information

Supplementary Table Legends.

Supplementary Table S1.

Supplementary Table S2.

Supplementary Table S3.

Supplementary Table S4.
