# Supplementary material for: Identification of eQTLs associated with lipid metabolism in Longissimus dorsi muscle of pigs with different genetic backgrounds
Source: Sci Rep. 2020 Jun 17;10:9845. doi: 10.1038/s41598-020-67015-4 (PMC7300017; doi:10.1038/s41598-020-67015-4)
Supplement: Supplementary file 1 — Supplementary Table Legends. [file 41598_2020_67015_MOESM1_ESM.docx]

**Identification of eQTLs associated with lipid metabolism in *Longissimus dorsi* muscle of pigs with different genetic backgrounds**

Lourdes Criado-Mesas^1^*, Maria Ballester^2^, Daniel Crespo-Piazuelo^1,3^, Anna Castelló^1,3^, Ana I. Fernández^4^ and Josep M. Folch^1,3^

^1^Departament de Genòmica Animal, Centre de Recerca en Agrigenòmica (CRAG), CSIC-IRTA-UAB-UB, Barcelona, Spain.

^2^Departament de Genètica i Millora Animal, Institut de Recerca y Tecnologia Agraroalimentàries (IRTA), Caldes de Montbui, Spain.

^3^Departament de Ciència Animal i dels Aliments, Facultat de Veterinària, UAB, Bellaterra, Spain.

^4^Departamento de Mejora Genética Animal, Instituto Nacional de Investigación y Tecnología Agraria y Alimentaria (INIA), Madrid, Spain.

* Corresponding author

E-mail: lourdes.criado@cragenomica.es (LCM)

**Supplementary Table Legends**

**Supplementary Table S1:** List of significant associated SNPs within eQTLs intervals for the 45-muscle gene expression study in 3BCs.

**Supplementary Table S2:** List of significant associated SNPs within eQTLs intervals for the 45-muscle gene expression study in each backcross independently.

**Supplementary Table S3:** Significant eQTLs found for the 45-muscle gene expression study in each backcross independently. Start and end positions refer to the eQTL interval and are based on Sscrofa 11.1 assembly. Gene annotation was performed considering one additional Mb at the start and at the end of the eQTL interval. SNPs column indicates the number of SNPs within the eQTL interval. For the *cis*-eQTLs regions only the analyzed gene was annotated as positional candidate gene.

**Supplementary Table S4:** Significant *trans*-eQTLs for the hotspot regions found in each backcross independently. Start and end positions refer to the eQTL interval and are based on Sscrofa 11.1 assembly. Gene annotation was performed considering one additional Mb at the start and at the end of the eQTL interval. SNPs column indicates the number of SNPs within the eQTL interval.
